# Global, regional, and national burden of early-onset ischemic heart disease: trends and projections among adults aged 15–44 years from 1990 to 2046

**DOI:** 10.3389/fcvm.2025.1653335

**Published:** 2025-08-29

**Authors:** Yan Wang, Xuezhen Sun, Xueqin Zhang, Xu Wang, Ju Li, Kai Wang

**Affiliations:** ^1^ Department of Rehabilitation, The Affiliated Huaian No.1 People’s Hospital of Nanjing Medical University, Huaian, China; ^2^ Department of Intervention, Huaian Second People’s Hospital, Huaian, China; ^3^ Department of Rheumatology and Immunology, The Affiliated Huaian No.1 People’s Hospital of Nanjing Medical University, Huaian, China

**Keywords:** early-onset ischemic heart disease, young adults, global burden of disease, epidemiological trends, sex disparities, regional variations, future burden

## Abstract

**Background:**

Ischemic heart disease (IHD) among young adults represents an emerging global health concern, yet comprehensive epidemiological assessments remain limited. This study aimed to quantify the global burden of early-onset IHD and project future trends through 2046.

**Methods:**

We analyzed data from the Global Burden of Disease (GBD) 2021 study, examining IHD burden among adults aged 15–44 years across 204 countries and territories from 1990 to 2021. Age-standardized rates were calculated using WHO standard population weights. Temporal trends were assessed using estimated annual percentage change (EAPC) methods. Age-Period-Cohort models were employed to project burden through 2046. DALYs were disaggregated into Years Lived with Disability (YLDs) and Years of Life Lost (YLLs).

**Results:**

Globally, age-standardized incidence rates increased modestly from 36.54 (95% UI: 21.69–54.31) per 100,000 in 1990 to 39.18 (95% UI: 23.32–58.20) per 100,000 in 2021, representing a 7.2% increase. Despite modest rate changes, absolute incident cases increased substantially from 1.26 millions to 2.17 millions. Age-standardized mortality rates declined significantly by 15.2%, from 12.21 (95% UI: 11.57–12.88) to 10.35 (95% UI: 9.68–11.04) per 100,000. Disaggregated DALYs analysis revealed divergent trends: YLDs rates increased (EAPC: 0.39%) while YLLs rates declined (EAPC: −0.66%), reflecting improved acute survival but growing chronic disease burden. Men consistently demonstrated higher burden across all measures, with male-to-female ratios ranging from 1.7:1 for incidence to 2.7:1 for mortality in the 40–44 years age group. Substantial regional heterogeneity was observed, with East Asia showing the steepest incidence increases (EAPC: 0.63%) while High-income North America demonstrated declining trends (EAPC: −2.4%). Central Europe achieved the most substantial improvements in both mortality decline (EAPC: −2.16%) and overall disease burden reduction (EAPC: −4.46%). Projections indicate continued increases in incidence and prevalence through 2046, with incident cases reaching 2.58 millions and prevalent cases reaching 12.7 millions globally.

**Conclusions:**

Early-onset IHD represents a growing global health challenge characterized by increasing incidence and prevalence but improving survival outcomes. The substantial sex and regional disparities, coupled with projected increases in absolute burden, underscore the urgent need for balanced strategies addressing both acute care improvements and long-term disability prevention.

## Introduction

Ischemic heart disease (IHD) remains the leading cause of mortality and morbidity worldwide, traditionally viewed as a disease of older adults. However, accumulating evidence suggests an alarming emergence of early-onset IHD among young adults, challenging conventional perceptions of cardiovascular disease epidemiology (1). The increasing recognition of myocardial infarction and other acute coronary syndromes in individuals under 45 years has prompted growing concern among clinicians and public health professionals, as this demographic was previously considered at low cardiovascular risk (2–4). Recent population-based studies from high-income countries have documented concerning trends in acute myocardial infarction among young adults, with incidence rates remaining stable or showing modest increases in several regions, contrasting with declining trends observed in older populations (5, 6).

The age range of 15–44 years was selected based on several considerations. First, the GBD database reports zero values for all IHD measures in the 0–14 years age group, reflecting the biological reality that clinical IHD is extremely rare in children. Second, while clinical definitions often use age cutoffs of <55 for men and <65 for women, epidemiological studies increasingly recognize that cardiovascular risk acceleration begins in the third and fourth decades of life (7). Third, the 15–44 years range captures the population during their most productive years, where IHD has the greatest socioeconomic impact through premature disability and mortality. This definition aligns with recent epidemiological studies focusing on “young adult” cardiovascular disease and allows for consistent global comparisons using standardized GBD methodology (8–10).

The etiology of early-onset IHD is multifactorial, encompassing traditional cardiovascular risk factors such as diabetes mellitus, hypertension, dyslipidemia, and smoking, alongside emerging risk factors including substance abuse, autoimmune conditions, and genetic predisposition (11–13). Contemporary lifestyle changes, particularly in developing nations undergoing rapid economic transition, have contributed to the earlier onset of metabolic syndrome and its associated cardiovascular complications (14). The global COVID-19 pandemic has further highlighted the vulnerability of young adults with cardiovascular disease, as emerging evidence suggests increased thrombotic risk and accelerated atherosclerosis among younger populations (15, 16).

Despite the clinical significance of early-onset IHD, comprehensive global analyses of its epidemiological burden remain limited. Previous studies have predominantly focused on single-country or regional analyses, often with restricted age ranges or limited temporal scope (17–19). The lack of standardized global surveillance data has hindered our understanding of worldwide patterns, temporal trends, and regional disparities in early-onset IHD burden.

The implications of early-onset IHD extend far beyond immediate clinical concerns. Young adults diagnosed with cardiovascular disease face decades of potential life lost, substantial healthcare utilization, and significant socioeconomic impact due to premature disability and reduced productivity (20, 21). The psychological burden associated with cardiovascular disease diagnosis in young adulthood, including anxiety, depression, and reduced quality of life, further compounds the individual and societal costs (22). From a healthcare system perspective, the management of early-onset IHD requires specialized approaches, including age-appropriate risk factor modification strategies, long-term medication adherence programs, and consideration of unique social circumstances such as family planning and career development.

Recent advances in global health monitoring, particularly through the Global Burden of Disease (GBD) initiative, have enabled comprehensive assessment of disease patterns across diverse populations and extended temporal periods. The GBD 2021 study provides an unprecedented opportunity to examine early-onset IHD burden systematically across 204 countries and territories, offering insights into global patterns, temporal trends, and regional disparities that were previously unavailable (23). Understanding these epidemiological patterns is crucial for informing evidence-based prevention strategies, optimizing healthcare resource allocation, and guiding policy development to address this emerging public health challenge.

Given the growing recognition of early-onset IHD as a significant health concern and the limited availability of comprehensive global analyses, this study aimed to provide a systematic examination of IHD burden among adults aged under 45 years from 1990 to 2021. Specifically, we sought to: (1) quantify global and regional trends in incidence, prevalence, mortality, and disability-adjusted life years; (2) identify geographical patterns and disparities in disease burden; (3) examine age and sex-specific patterns within the young adult population; and (4) project future burden trends through 2046 using advanced modeling techniques. These findings will contribute essential evidence for developing targeted prevention strategies and informing healthcare policy decisions to address the growing challenge of early-onset ischemic heart disease globally.

## Methods

### Data source and study design

This study analyzed data from the Global Burden of Disease (GBD) 2021 study, examining ischemic heart disease (IHD) burden among young adults aged under 45 years from 1990 to 2021. The analysis encompassed epidemiological indicators including incidence, prevalence, mortality, and disability-adjusted life years (DALYs) across 204 countries and territories, 27 regions, and five socio-demographic index (SDI) levels. DALYs were analyzed both as composite measures and disaggregated into Years Lived with Disability (YLDs) and Years of Life Lost (YLLs) to capture distinct patterns in morbidity and mortality burden. Given that the GBD database reports zero values for all IHD measures (incidence, prevalence, deaths, and DALYs) in the 0–14 years age group, the effective study population comprised individuals aged 15–44 years, representing the true early-onset IHD population. The study population was initially stratified into nine age groups: <5 years, 5–9 years, 10–14 years, 15–19 years, 20–24 years, 25–29 years, 30–34 years, 35–39 years, and 40–44 years. However, since all epidemiological measures showed zero values for children aged 0–14 years, the analytical focus was concentrated on six age groups spanning 15–44 years. Age-standardized rates (ASRs) were calculated using the World Health Organization (WHO) 2000–2025 world standard population as the reference standard. For the under-45 population analysis, the WHO standard population weights were extracted for the 0–44 years age range and subsequently normalized to ensure appropriate standardization for the specific population of interest, with the 0–14 years weight combined as a single category despite having zero disease burden.

### Statistical analysis

Data extraction, processing, and statistical analysis procedures, including estimated annual percentage change (EAPC) calculations and Age-Period-Cohort (APC) modeling methodologies, were conducted following standardized approaches as detailed in our previous work (24, 25). ASRs were computed using the direct standardization method, accounting for the unique age distribution of early-onset IHD. For each epidemiological measure, the age-specific rates were weighted by the corresponding WHO standard population weights using the formula: ASR = Σ(rate_i × weight_i), where rate_i represents the age-specific rate for age group i, and weight_i represents the WHO standard population weight for the corresponding age group. Since the 0–14 years age groups contributed zero cases but non-zero population weights, these were maintained in the standardization process to ensure comparability with other studies while recognizing that the actual disease burden originated entirely from the 15–44 years population.

Temporal trends were assessed using the EAPC method implemented across all geographic regions and countries. For each region and country, EAPC was calculated by fitting a log-linear regression model: ln(ASR) = α + βt + ε, where t represents the calendar year, and β represents the regression coefficient. The EAPC was calculated as: EAPC = (e^β - 1) × 100%, with 95% uncertainty intervals (UI) derived from the standard error of the regression coefficient. This analysis provided insights into whether IHD burden among young adults was increasing, decreasing, or remaining stable over the 31-year study period.

Future burden projections from 2022 to 2046 were generated using APC models implemented through the nordpred package in R software. Key model assumptions include: (1) log-linear age effects within each age group; (2) smooth temporal trends that may change according to predefined cutoff points (cuttrend parameters: 0, 0.25, 0.5, 0.75, 0.75); (3) birth cohort effects following smooth patterns; and (4) stable diagnostic criteria and case definitions throughout the study period. Model validation was performed using out-of-sample prediction analysis. The final time period (2017–2021) was withheld from model training and used for validation testing. The trained model predicted the held-out period, and performance was assessed using mean absolute percentage error (MAPE) and root mean square error (RMSE):

$$\begin{array}{rl} & \mathrm{MAPE}\;=\;(1/n)\;\times \Sigma |\left((\mathrm{predicted}\;\text{-}\;\mathrm{actual})/\mathrm{actual}\right)|\;\times 100\text{\%}\\ & \mathrm{RMSE}\;=\;\sqrt{\left((1/n)\;\times \Sigma {\left(\mathrm{predicted}\;\text{-}\;\mathrm{actual}\right)}^{2}\right)} \end{array}$$

Uncertainty quantification was implemented through bootstrap resampling with 100 iterations. For each bootstrap iteration, Poisson noise was added to observed case counts to simulate natural variation in disease occurrence. The APC model was fitted to each bootstrap sample, generating prediction distributions for 2022–2046. The 95% UI were calculated as the 2.5th and 97.5th percentiles of the bootstrap distribution. This approach captures both parameter uncertainty and natural variation in disease occurrence. Data processing and all statistical analyses were conducted using R (version 4.3.2) with specialized packages including data.table, dplyr, readr, ggplot2, nordpred, BAPC, and gridExtra, with statistical significance set at *p* < 0.05.

## Results

### Global trends in IHD among population under 45 years

**Figure 1:**
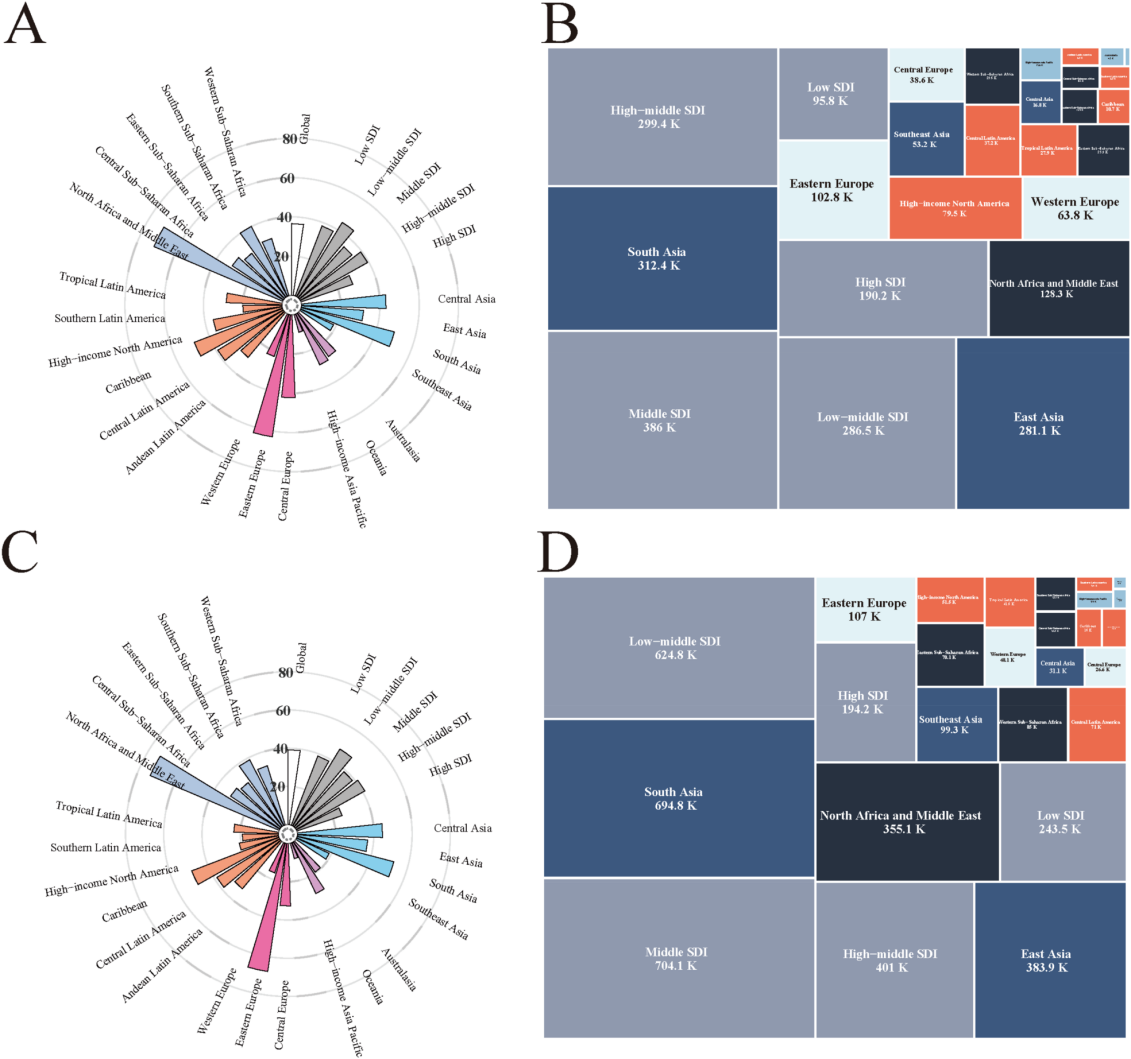
Global epidemiological characteristics of early-onset IHD. **(A)** Regional distribution of ASIR of early-onset IHD in 1990: Radial bar chart showing the ASIRs across different regions and countries, expressed per 100,000 population. **(B)** Incident cases of early-onset IHD in 1990: Treemap visualization displaying the distribution of incident cases across different socio-demographic index (SDI) regions, with values presented in thousands. Middle SDI regions (386K) and South Asia (312.4K) had the highest number of incident cases. **(C)** Regional distribution of ASIR of early-onset IHD in 2021: Compared to 1990, showing the temporal changes in ASIRs across regions, expressed per 100,000 population. **(D)** Incident cases of early-onset IHD in 2021: Treemap showing the distribution of incident cases across different SDI regions in 2021, with South Asia (694.8K) and low-middle SDI regions (624.8K) demonstrating substantial increases in case numbers. IHD, ischemic heart disease; SDI, socio-demographic index; ASIR, age-standardized incidence rate; K, thousands of cases.

Globally, the age-standardized incidence rate (ASIR) of IHD in the population under 45 years showed a modest increase from 36.54 (95% UI: 21.69–54.31) per 100,000 in 1990 to 39.18 (95% UI: 23.32–58.20) per 100,000 in 2021, representing a 7.2% increase over the 31-year period (Figure 1). Regionally, North Africa and Middle East had the highest ASIR in 2021 at 73.30 (95% UI: 45.30–106.26) per 100,000, while High-income Asia Pacific had the lowest at 8.31 (95% UI: 4.08–13.69) per 100,000. Despite the modest increase in ASRs, the absolute number of incident cases increased substantially from 1.26 (95% UI: 0.74–1.89) millions in 1990 to 2.17 (95% UI: 1.29–3.22) millions in 2021, representing a 72.3% increase. South Asia had the highest absolute incident cases in 2021 with 694,789 cases, while Australasia had the lowest with 4,208 cases.

The global age-standardized prevalence rate (ASPR) increased from 195.50 (95% UI: 156.02–242.38) per 100,000 in 1990 to 210.56 (95% UI: 162.02–271.24) per 100,000 in 2021, representing a 7.7% increase (Supplementary Figure S1). Eastern Europe showed the highest ASPR in 2021 at 372.92 (95% UI: 273.89–496.60) per 100,000, while High-income Asia Pacific had the lowest at 67.86 (95% UI: 51.77–86.16) per 100,000. The absolute number of prevalent cases nearly doubled from 6.67 (95% UI: 5.31–8.28) millions in 1990 to 11.67 (95% UI: 8.98–15.04) millions in 2021, representing a 75.1% increase. South Asia had the highest prevalent cases in 2021 with 3.28 millions cases, while Australasia had the lowest with 22,169 cases.

In contrast to incidence and prevalence trends, the global age-standardized mortality rate (ASMR) decreased significantly from 12.21 (95% UI: 11.57–12.88) per 100,000 in 1990 to 10.35 (95% UI: 9.68–11.04) per 100,000 in 2021, representing a 15.2% reduction (Supplementary Figure S2). South Asia had the highest ASMR in 2021 at 16.99 (95% UI: 15.24–18.64) per 100,000, while High-income Asia Pacific had the lowest at 1.79 (95% UI: 1.65–1.95) per 100,000. Despite declining ASRs, the absolute number of deaths increased from 270,712 (95% UI: 256,155–285,894) in 1990 to 364,979 (95% UI: 341,257–389,131) in 2021, representing a 34.8% increase. South Asia had the highest absolute deaths in 2021 with 145,395 deaths, while Australasia had the lowest with 314 deaths.

The global age-standardized DALYs rate (ASDR) demonstrated a similar declining pattern, decreasing from 419.56 (95% UI: 397.24–442.89) per 100,000 in 1990 to 357.37 (95% UI: 334.33–381.08) per 100,000 in 2021, indicating a 14.8% reduction (Supplementary Figure S3). South Asia had the highest ASDR in 2021 at 580.41 (95% UI: 521.15–636.60) per 100,000, while High-income Asia Pacific had the lowest at 63.30 (95% UI: 58.67–68.67) per 100,000. However, the absolute number of DALYs increased from 14.78 (95% UI: 13.98–15.62) millions in 1990 to 19.75 (95% UI: 18.48–21.06) millions in 2021, representing a 33.6% increase. South Asia had the highest absolute DALYs in 2021 with 7.84 millions, while Australasia had the lowest with 16,606.

The disaggregated analysis of DALYs components revealed distinct patterns for YLDs and YLLs. Globally, age-standardized YLDs rates increased from 6.58 (95% UI: 4.16–9.82) per 100,000 in 1990 to 7.24 (95% UI: 4.55–10.76) per 100,000 in 2021, while YLL rates declined from 651.6 (616.5–688.3) to 553.4 (516.9–590.1) per 100,000 (Supplementary Figures S4, S5). The absolute burden showed substantial increases, with YLDs rising from 0.15 (95% UI: 0.09–0.22) millions to 0.26 (95% UI: 0.16–0.38) million, while YLLs increased modestly from 14.63 (95% UI: 13.83–15.48) millions to 19.5 (95% UI: 18.22–20.79) millions.

### Temporal trends and regional variations

**Figure 2:**
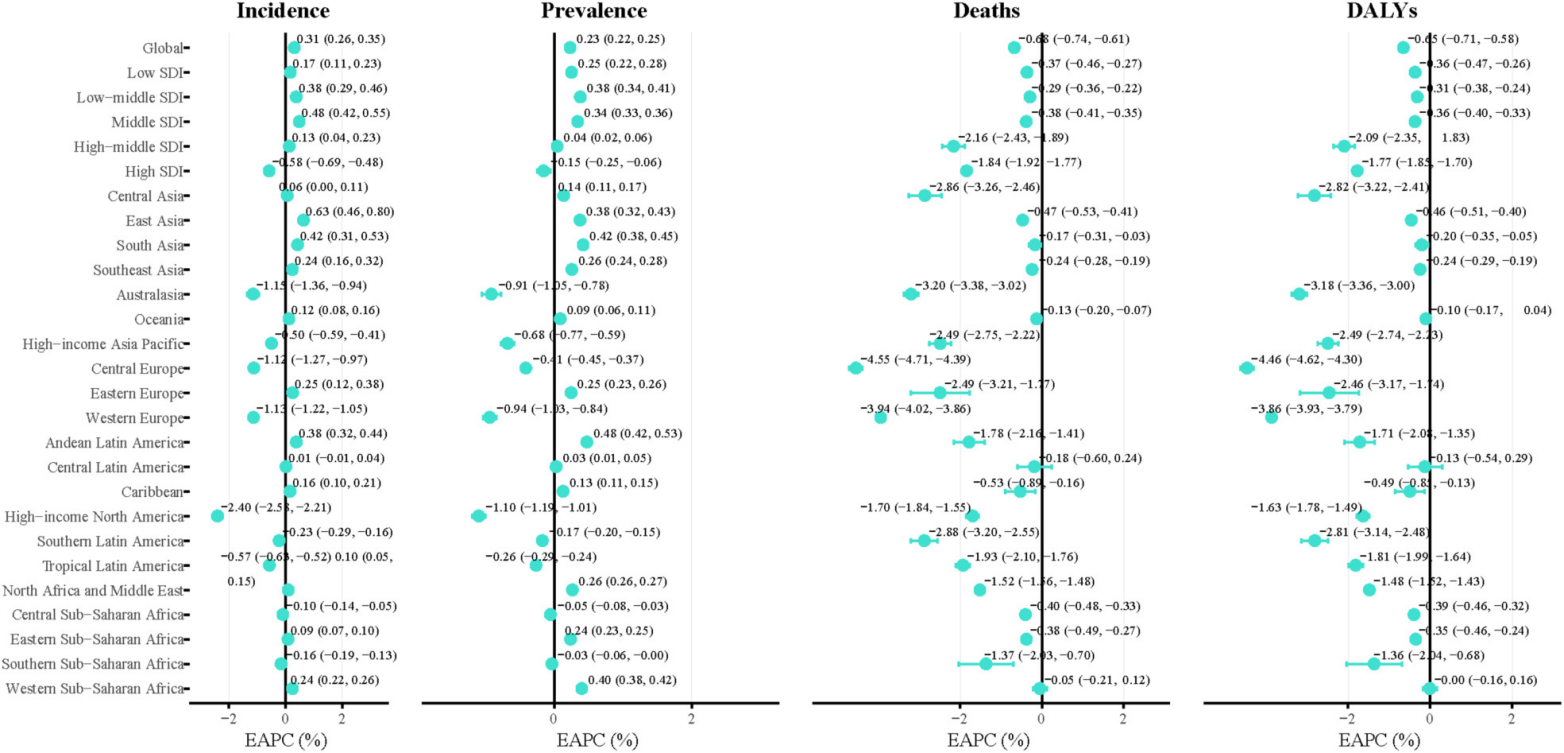
Temporal trends in early-onset IHD burden by region and SDI level from 1990 to 2021. Forest plots showing the EAPC with 95% UIs for ASIR, ASPR, ASMR, and ASDR across different geographic regions and SDI levels. Positive EAPC values indicate increasing trends, while negative values indicate declining trends. The global EAPC was 0.31% for incidence, 0.23% for prevalence, −0.61% for mortality, and −0.45% for DALYs. IHD, ischemic heart disease; SDI, socio-demographic index; EAPC, estimated annual percentage change; UI, uncertainty intervals; ASIR, age-standardized incidence rate; ASPR, age-standardized prevalence rate; ASMR, age-standardized mortality rate; DALYs, disability-adjusted life years; ASDR, age-standardized DALYs rate.

The temporal analysis revealed substantial regional heterogeneity in IHD trends among young adults from 1990 to 2021 (Figure 2). Globally, the ASIR showed a modest increasing trend with an EAPC of 0.31% (95% UI: 0.26, 0.35). The most pronounced increases were observed in East Asia (EAPC: 0.63%, 95% UI: 0.46–0.8), while several regions demonstrated declining trends, notably High-income North America (EAPC: −2.4%, 95% UI: −2.58, −2.21).

The global ASPR demonstrated a slight upward trend with an EAPC of 0.23% (95% UI: 0.22, 0.25) annually. Most regions showed increasing prevalence rates, with the highest increases in Andean Latin America (EAPC: 0.48%, 95% UI: 0.42, 0.53) and Low-middle SDI countries (EAPC: 0.34%, 95% UI: 0.33, 0.36). Notable exceptions included High-income North America (EAPC: −1.1%, 95% UI: −1.19, −1.01) and Western Europe (EAPC: −0.94%, 95% UI: −1.03, −0.84), which exhibited declining prevalence trends.

Encouragingly, both global ASMR and ASDR demonstrated significant declining trends, with EAPCs of −0.61% (95% UI: −0.74, −0.61) and −0.45% (95% UI: −0.71, −0.58) annually, respectively. This pattern of mortality and disease burden reduction was consistently observed across all regions worldwide. Central Europe exhibited the most substantial improvements in both measures, achieving the steepest mortality decline (EAPC: −2.16%, 95% UI: −4.71, −4.39) and the largest reduction in overall disease burden (EAPC: −4.46%, 95% UI: −4.62, −4.3), demonstrating the most pronounced progress in addressing early-onset IHD burden globally.

Regional analysis of DALY components demonstrated heterogeneous patterns. Globally, YLD rates increased (EAPC: 0.39%, 95% UI: 0.26–0.52) while YLL rates declined (EAPC: −0.66%, 95% UI: −0.78–0.54). For YLDs, the largest increases were observed in East Asia (EAPC: 1.16%, 95% UI: 0.98–1.35), while Australasia showed declining trends (EAPC: −1.44%, 95% UI: −1.52–1.37). YLLs patterns mirrored mortality trends, with the steepest declines in Central Europe (EAPC: −4.53%, 95% UI: −4.69–4.37) and Western Europe (EAPC: −3.95%, 95% UI: −4.03–3.87) (Supplementary Figure S6).

### Global distribution patterns and country-level analysis

**Figure 3:**
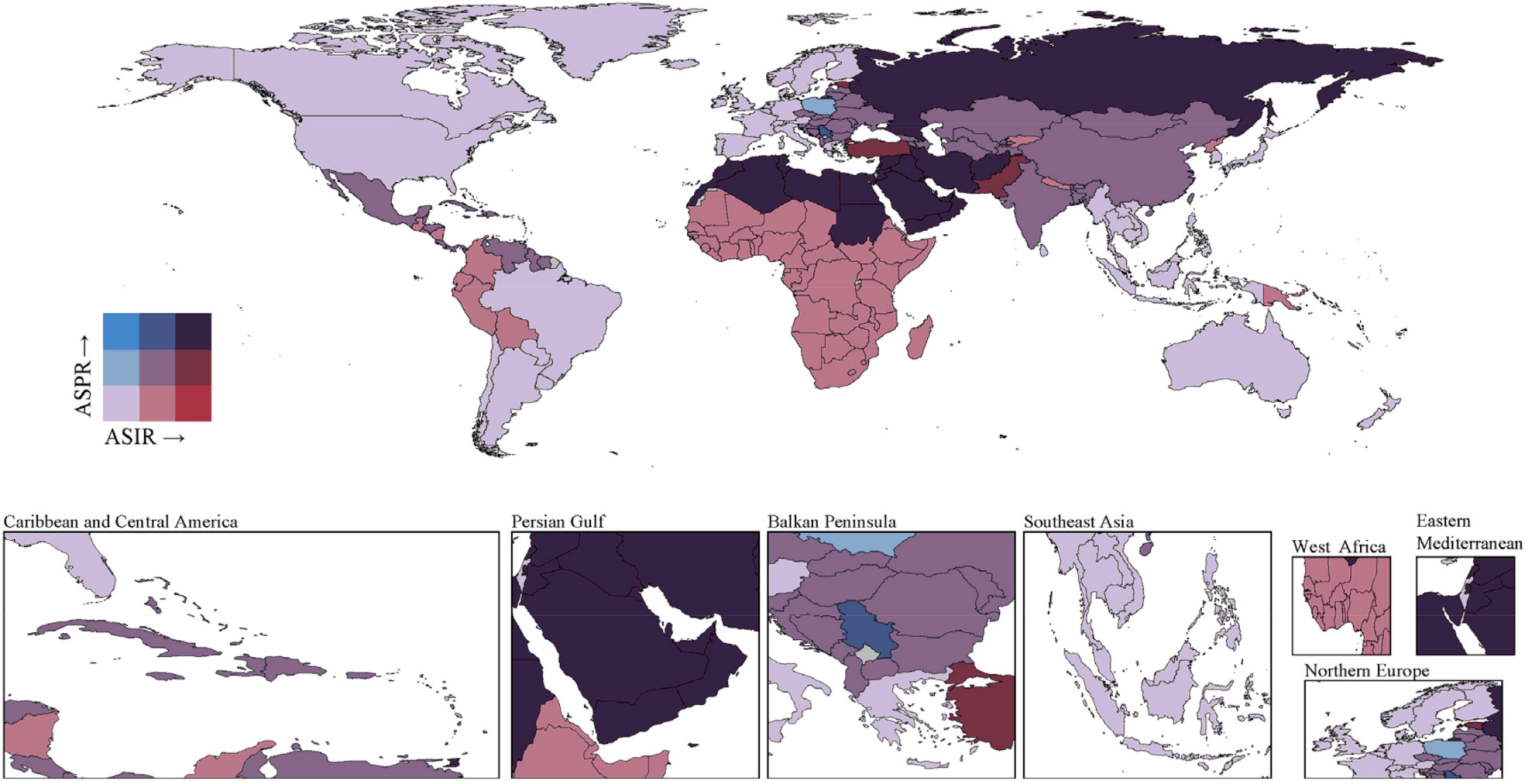
Bivariate choropleth map showing the global distribution of ASIR and ASPR of early-onset IHD in 2021. The color gradient represents the combination of incidence and prevalence patterns across countries. Gulf countries including the United Arab Emirates, Kuwait, and Saudi Arabia exhibited high incidence-high prevalence patterns, with the United Arab Emirates showing an ASIR of 80.21 per 100,000 and ASPR of 394.05 per 100,000. In contrast, developed European countries such as Sweden, Norway, and Denmark demonstrated low incidence-low prevalence patterns, with Sweden recording an ASIR of 16.42 per 100,000 and ASPR of 88.65 per 100,000. Eastern European countries displayed disproportionate relationships with relatively lower incidence but higher prevalence rates. IHD, ischemic heart disease; ASIR, age-standardized incidence rate; ASPR, age-standardized prevalence rate.

The global distribution of IHD among young adults in 2021 demonstrated significant geographical disparities in ASRs of incidence and prevalence. Bivariate map analysis revealed distinct combination patterns across different countries and regions (Figure 3). Gulf countries, including the United Arab Emirates, Kuwait, and Saudi Arabia, exhibited a high incidence-high prevalence pattern, with the United Arab Emirates showing an incidence rate of 80.21 per 100,000 person-years and a prevalence rate of 394.05 per 100,000 population. In contrast, developed European countries such as Sweden, Norway, and Denmark demonstrated a low incidence-low prevalence pattern, with Sweden recording an incidence rate of 16.42 per 100,000 person-years and a prevalence rate of 88.65 per 100,000 population.

Several countries exhibited disproportionate relationships between incidence and prevalence rates. Eastern European countries, including Hungary and Estonia, displayed relatively lower incidence rates (33.81 and 55.32 per 100,000 person-years, respectively) but substantially higher prevalence rates (263.62 and 301.90 per 100,000 population, respectively). Conversely, sub-Saharan African countries such as Ethiopia and Niger presented moderate incidence rates but relatively lower prevalence rates, possibly reflecting underdiagnosis or higher early mortality rates.

Bivariate analysis of mortality and disease burden (DALYs) revealed substantial regional inequalities in health losses caused by ischemic heart disease among young adults (Supplementary Figure S7). Pacific Island nations demonstrated the most severe disease burden, with Nauru recording a mortality rate of 78.09 per 100,000 person-years and DALYs of 2,610.35 per 100,000 person-years. Middle Eastern countries exhibited high mortality characteristics, with Egypt recording a mortality rate of 29.27 per 100,000 person-years and DALYs of 1,040.66 per 100,000 person-years. Western European developed countries maintained low levels in both mortality and disease burden dimensions, with Sweden recording a mortality rate of 0.82 per 100,000 person-years and DALYs of 30.32 per 100,000 person-years.

### Age and sex patterns

**Figure 4:**
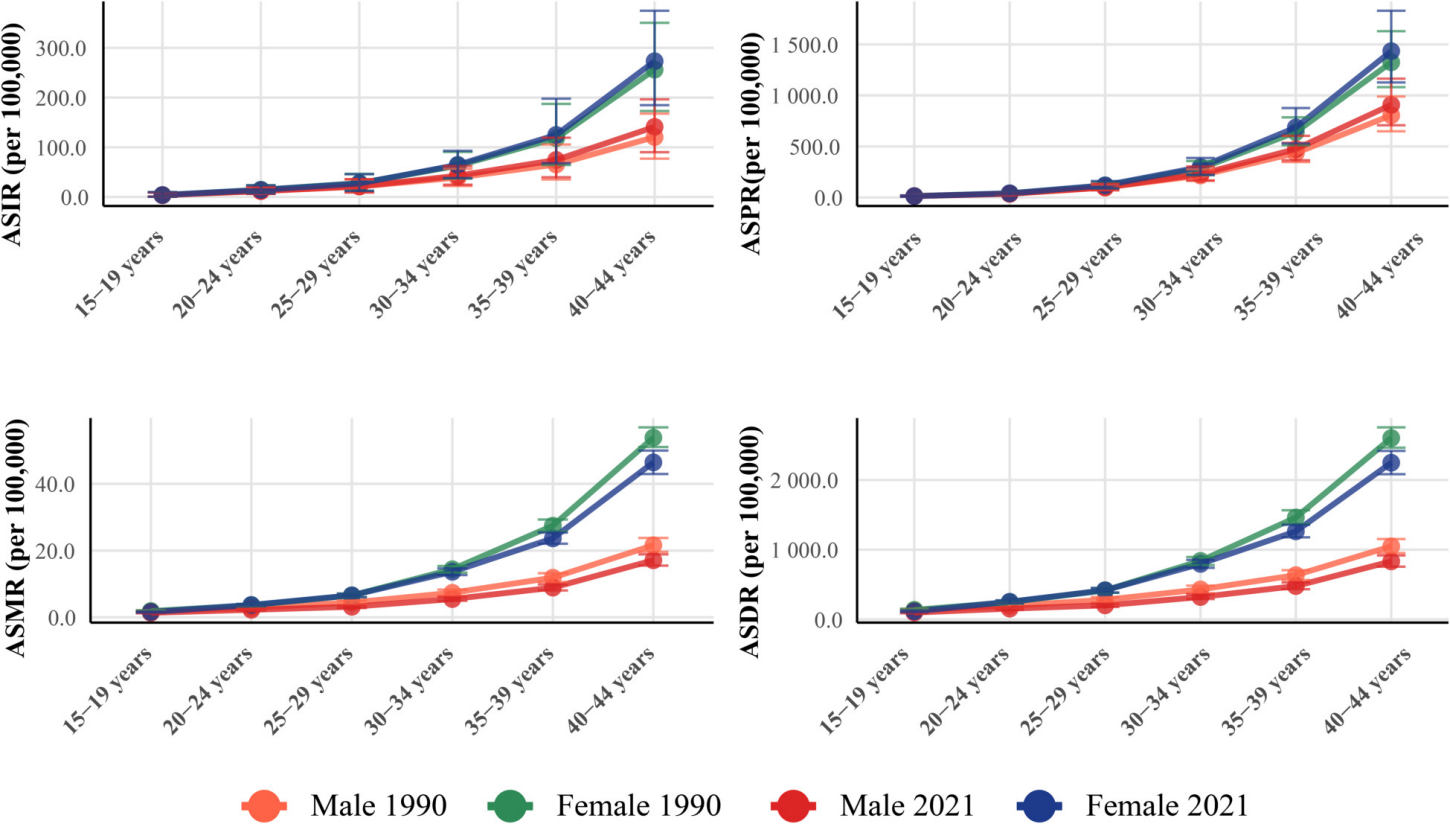
Age and sex patterns of early-onset IHD burden in 1990 and 2021. ASRs with 95% UIs for ASIR, ASPR, ASMR, and ASDR by sex across six age groups from 15 to 44 years in 1990 and 2021. All measures demonstrated pronounced age gradients with exponential increases in older age groups. In 2021, female incidence rates increased from 3.64 per 100,000 in the 15–19 years group to 141.02 per 100,000 in the 40–44 years group (39-fold increase), while male rates escalated from 3.93 to 273.10 per 100,000 (69-fold increase). Mortality rates showed the steepest age gradient, with male-to-female ratios reaching 2.7:1 in the 40–44 years group. Men consistently demonstrated higher burden across all measures and age groups, with the sex disparity widening progressively with advancing age. IHD, ischemic heart disease; UI, uncertainty intervals; ASIR, age-standardized incidence rate; ASPR, age-standardized prevalence rate; ASMR, age-standardized mortality rate; ASDR, age-standardized DALY rate; DALYs, disability-adjusted life years.

The age-specific analysis revealed pronounced age gradients for all epidemiological measures, with the steepest increases observed in older age groups within the young adult population (Figure 4). In 2021, female incidence rates increased from 3.64 (95% UI: 0.66–8.95) per 100,000 in the 15–19 years age group to 141.02 (95% UI: 90.13–196.45) per 100,000 in the 40–44 years age group, representing a 39-fold increase. Male incidence rates showed an even more dramatic escalation, from 3.93 (95% UI: 0.63–9.78) per 100,000 in the 15–19 years group to 273.10 (95% UI: 184.67–374.68) per 100,000 in the 40–44 years group, indicating a 69-fold increase. The male-to-female incidence rate ratio increased progressively with age, from approximately 1.1:1 in the 15–19 years group to 1.9:1 in the 40–44 years group. The increase was more pronounced among females (79.5% increase from 441,363 to 792,496 cases) compared to males (68.4% increase from 817,377 to 1,376,754 cases), though males continued to bear a disproportionately higher absolute burden (Supplementary Figure S8). The male-to-female incidence ratio showed a slight convergence from 1.85:1 in 1990 to 1.74:1 in 2021, suggesting a narrowing but persistent gender gap. Female prevalence increased by 82.2% (from 2,387,309 to 4,349,511 cases), while male prevalence increased by 78.3% (from 4,032,275 to 7,187,817 cases).

Mortality rates exhibited the most pronounced age gradient among all measures. Female mortality rates increased from 1.35 (95% UI: 1.20–1.50) per 100,000 in the 15–19 years group to 17.09 (95% UI: 15.48–18.87) per 100,000 in the 40–44 years group, representing a 13-fold increase. Male mortality rates showed a more dramatic escalation, from 1.58 (95% UI: 1.41–1.76) per 100,000 in the youngest group to 46.42 (95% UI: 42.94–49.97) per 100,000 in the oldest group, indicating a 29-fold increase. The male-to-female mortality ratio increased substantially with age, reaching 2.7:1 in the 40–44 years group in 2021. Deaths from early-onset IHD increased by 32.5% globally, from 270,057 to 357,938 deaths. The increase was more pronounced among males (37.9% increase from 189,419 to 261,252 deaths) compared to females (19.9% increase from 80,638 to 96,686 deaths). This gender disparity in mortality trends contributed to a widening of the male-to-female death ratio over the study period. Male DALYs increased by 41.2% (from 9,958,867 to 14,058,479 years), while female DALYs increased by 38.9% (from 4,820,371 to 6,693,225 years). The substantial increase in DALYs reflects both the rising incidence and the premature nature of early-onset IHD, which disproportionately affects individuals during their most productive years.

Age-specific analysis of DALYs components revealed pronounced patterns across the 15–44 years age range. In 2021, female YLDs increased dramatically from 1,563 years in the 15–19 years group to 37,126 years in the 40–44 years group, representing a 24-fold increase, while female YLLs rose from 297,663 years to 2,028,627 years, indicating a 7-fold increase. Male YLDs showed an even steeper gradient, escalating from 2,084 years in the youngest group to 64,653 years in the oldest group (31-fold increase), while male YLLs increased from 366,712 years to 5,603,291 years (15-fold increase). The male-to-female ratios demonstrated substantial age-related disparities: YLDs ratios increased from 1.3:1 in the 15–19 years group to 1.7:1 in the 40–44 years group, while YLLs ratios expanded from 1.2:1 to 2.8:1 across the same age range (Supplementary Figures S9, S10).

### Future projections

**Figure 5:**
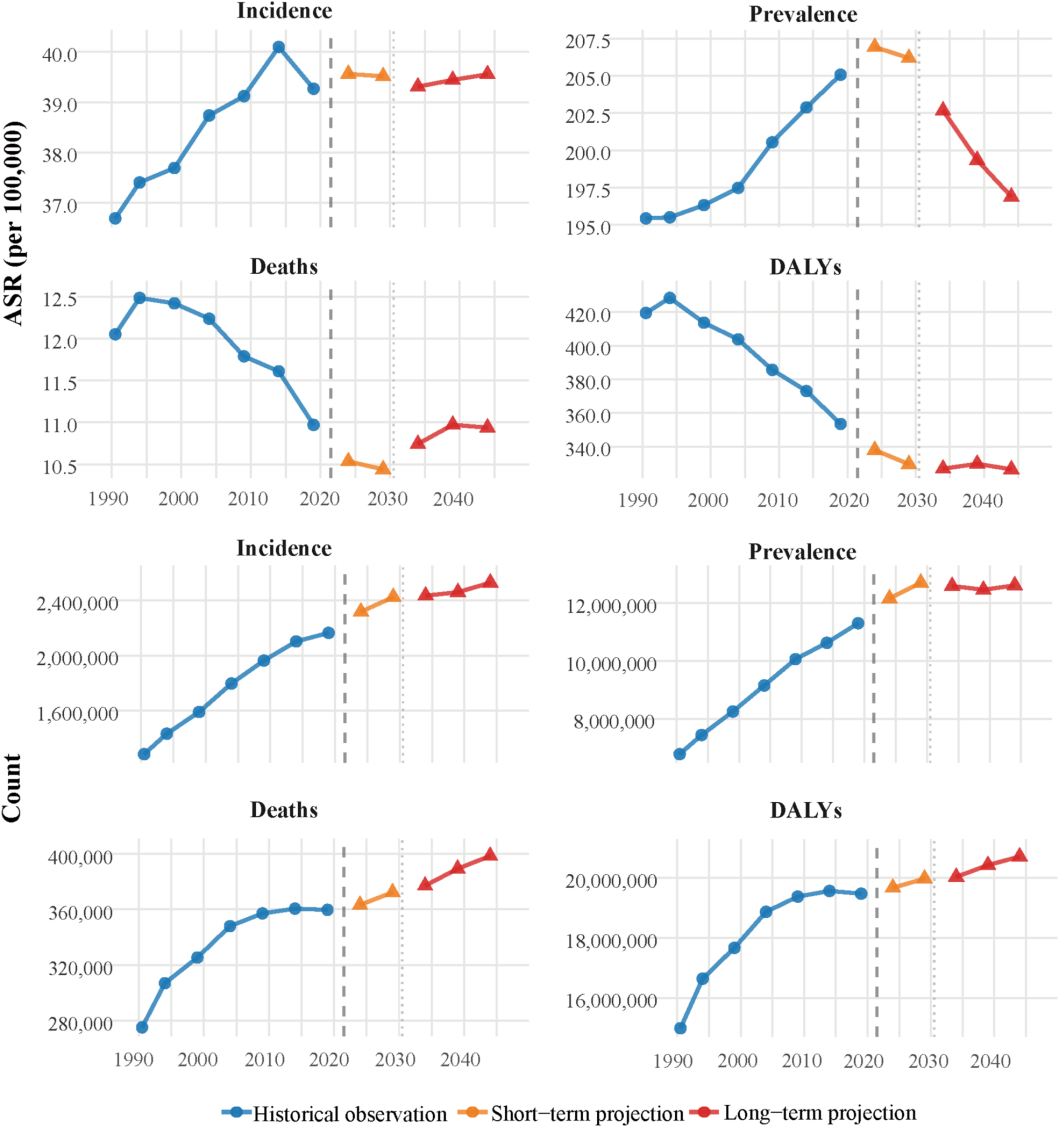
Historical trends and future projections of early-onset IHD burden from 1990 to 2046. APC model projections showing historical observations (1990–2021) and future trends through 2046 for both age-standardized rates (upper panels) and absolute counts (lower panels). ASIRs are projected to continue rising to 40.3 (95% UI: 40–40.6) per 100,000 by 2046, with absolute incident cases reaching 2.58 (95% UI: 2.56–2.59) millions globally. ASPRs are projected to reach 198.1 (95% UI: 197.5–198.6) per 100,000 by 2046, corresponding to 12.7 (95% UI: 12.66–12.73) millions prevalent cases worldwide. ASMRs show a continued declining trajectory to 11.4 (95% UI: 11.3–11.5) per 100,000 by 2046, though absolute deaths are expected to increase to 0.42 (95% UI: 0.41–0.42) million cases. ASDRs are projected to stabilize around 337.7 (95% UI: 337.3–338.4) per 100,000 by 2046, with total projected DALYs reaching 21.43 (95% UI: 21.4–21.47) millions years. The vertical dashed line at 2021 separates historical observations from projections, with short-term (2022–2031) and long-term (2032–2046) projection periods indicated by different colors. The model validation demonstrated satisfactory predictive performance with MAPE of 3.76% for ASIRs (correlation coefficient: 0.995), 2.06% for ASPRs (correlation coefficient: 0.996), 7.14% for ASMRs (correlation coefficient: 0.998), and 4.24% for ASDRs (correlation coefficient: 0.997) using out-of-sample prediction (training: 1990–2011, validation: 2012–2021). The UIs shown in the projections capture both parameter uncertainty and natural variation in disease occurrence through bootstrap resampling methods. IHD, ischemic heart disease; APC, age-period-cohort; ASIR, age-standardized incidence rate; ASPR, age-standardized prevalence rate; ASMR, age-standardized mortality rate; ASDR, age-standardized DALY rate; DALYs, disability-adjusted life years; UI, uncertainty intervals; MAPE, mean absolute percentage error; RMSE, root mean square error.

APC models projected concerning upward trends in IHD burden among young adults from 2022 to 2046 (Figure 5). The ASIR is projected to continue rising to 40.3 (95% UI: 40–40.6) per 100,000 person-years by 2046, with the absolute number of incident cases reaching 2.58 (95% UI: 2.56–2.59) millions globally. ASPRs are projected to reach 198.1 (95% UI: 197.5–198.6) per 100,000 population by 2046, corresponding to approximately 12.7 (95% UI: 12.66–12.73) millions prevalent cases worldwide. Despite increasing incidence and prevalence, mortality projections revealed a continued declining trajectory, with the ASMRs projected to be 11.4 (95% UI: 11.3–11.5) per 100,000 person-years in 2046, though absolute deaths are expected to increase to 0.42 (95% UI: 0.41–0.42) million. ASDRs are projected to stabilize around 337.7 (95% UI: 337.3–338.4) per 100,000 person-years by 2046, with total projected DALYs reaching 21.43 (95% UI: 21.4–21.47) millions years. YLDs rates are projected to continue rising to 333.5 (95% UI: 333–334) per 100,000 by 2046, corresponding to approximately 21.2 (95% UI: 21.1–21.2) millions years worldwide, while YLLs rates are projected to reach 4.3 (95% UI: 4.3–4.4) per 100,000 by 2046, with absolute YLLs reaching 0.27 (95% UI: 0.27–0.28) millions years globally (Supplementary Figure S1^1^).

## Discussion

This comprehensive analysis of the Global Burden of Disease 2021 data reveals a concerning epidemiological transition in ischemic heart disease among young adults aged 15–44 years, characterized by rising incidence and prevalence rates coupled with declining mortality rates. The 7.2% increase in global ASIRs from 1990 to 2021, alongside the projected continuation of this upward trend through 2046, underscores the emerging threat of early-onset IHD as a significant public health challenge. These findings align with recent observations from population-based studies demonstrating increasing trends in acute myocardial infarction among younger adults, particularly in high-income countries (7, 18, 26). The substantial 72.3% increase in absolute incident cases over the study period, despite modest changes in age-standardized rates, reflects the dual impact of population growth and demographic transitions, highlighting the urgent need for targeted prevention strategies in this vulnerable population.

The pronounced male predominance observed across all epidemiological measures, with male-to-female ratios ranging from 1.7:1 for incidence to 2.7:1 for mortality in the 40–44 years age group, reflects well-established biological and behavioral risk factor differences. The persistent and widening male predominance reflects complex interactions of biological, behavioral, and sociocultural factors (27). Biologically, men experience earlier onset of atherosclerotic risk factors, including adverse lipid profiles and insulin resistance (4). Behaviorally, young men demonstrate higher rates of smoking, alcohol consumption, and risky dietary patterns, while exhibiting lower healthcare utilization and delayed care-seeking behaviors (8, 26, 28). Sociocultural factors, including occupational stress and traditional masculine norms that discourage preventive health behaviors, further amplify risk (29). These insights suggest that sex-specific intervention strategies should include: targeted screening programs for men aged 30–44 years, workplace-based prevention programs, and culturally adapted health promotion campaigns that address masculine health beliefs while promoting early identification and management of cardiovascular risk factors.

The disaggregated analysis of YLDs and YLLs provides critical insights into the evolving burden of early-onset IHD. The increasing YLD rates (EAPC: 0.39%) coupled with declining YLL rates (EAPC: −0.66%) reflect a epidemiological transition characterized by improved acute survival but growing chronic disease burden. This pattern suggests that while medical advances have reduced premature mortality, the absolute numbers of young adults living with IHD and its associated disabilities continue to rise, emphasizing the importance of both acute care improvements and long-term disability prevention strategies.

The substantial regional heterogeneity in temporal trends reveals striking disparities in early-onset IHD burden globally, reflecting complex interplay between socioeconomic development, healthcare systems, and population-level risk factors. The concerning increases in East Asia (EAPC: 0.63%) contrast sharply with declining trends in High-income North America (EAPC: −2.4%), likely reflecting differential implementation of population-level prevention strategies. High-income North America's declining trends coincide with comprehensive tobacco control policies, widespread adoption of Mediterranean dietary patterns, and universal healthcare systems facilitating early risk factor management. Conversely, the concerning increases in East Asia may reflect the obesity epidemic, increased prevalence of diabetes and metabolic syndrome, and healthcare access disparities despite advanced medical technology (14, 30, 31). The high burden observed in Gulf countries, with the United Arab Emirates demonstrating incidence rates exceeding 80 per 100,000 person-years, reflects rapid epidemiological transition associated with economic development, urbanization, adoption of Western dietary patterns, increased sedentary behavior, and high prevalence of diabetes and obesity. In contrast, the disproportionate relationship between incidence and prevalence in sub-Saharan African countries may indicate significant challenges in case detection, healthcare access, and survival, alongside the potential influence of younger population demographics and protective effects from traditional dietary patterns and higher physical activity levels. This pattern highlights the urgent need for strengthened cardiovascular care systems in these regions (32, 33).

The encouraging 15.2% reduction in global ASMRs, alongside the 14.8% decline in DALY rates, demonstrates significant progress in acute cardiovascular care and secondary prevention strategies over the past three decades. These improvements likely reflect advances in emergency cardiovascular interventions, including widespread implementation of primary percutaneous coronary intervention, evidence-based pharmacotherapy, and comprehensive cardiac rehabilitation programs (34, 35). The projected continuation of mortality decline through 2046, despite rising incidence, suggests that healthcare systems are adapting to manage the increasing burden of early-onset IHD more effectively. However, the substantial increases in absolute numbers of deaths and DALYs underscore that population-level prevention efforts must intensify to address the growing absolute burden.

The APC model projections indicating continued increases in incidence and prevalence through 2046, with absolute cases reaching 2.5 millions and 12.6 millions respectively, represent a significant challenge for global health systems. The stabilization of DALY rates around 326.5 per 100,000 person-years by 2046 suggests a potential equilibrium between improving survival and increasing disease occurrence, but the projected 20.7 millions total DALYs highlight the substantial societal impact. These projections align with recent modeling studies predicting increasing cardiovascular disease burden in younger populations, particularly in regions undergoing rapid economic transition (36, 37). The findings emphasize the critical importance of primordial prevention strategies targeting traditional risk factors, including tobacco control, healthy diet promotion, physical activity enhancement, and management of emerging risk factors such as air pollution and psychosocial stress.

Several important limitations should be acknowledged in interpreting these findings. The GBD methodology relies heavily on modeling approaches that may introduce uncertainty, particularly in low-resource settings with limited epidemiological surveillance systems. For example, in sub-Saharan African countries, potential underdiagnosis due to limited healthcare access may lead to underestimation of true disease burden, while in high-income countries, more sensitive diagnostic methods may capture milder cases, potentially inflating incidence rates. Data sparsity in some regions requires extrapolation from neighboring countries or regions with similar socio-demographic profiles, which may not accurately reflect local disease patterns. Additionally, cause-of-death misclassification, particularly in regions without standardized death certification systems, may affect mortality estimates, and these limitations likely contribute to the substantial regional variations observed. The zero burden in the 0–14 years age group, while reflecting biological reality, may limit direct comparability with studies using different age definitions for young adults. The analysis could not account for emerging risk factors such as environmental exposures, social determinants, or genetic predisposition that may contribute to early-onset IHD. Furthermore, temporal changes in diagnostic criteria, clinical practice patterns, and healthcare system capacity over the 31-year study period may influence trend interpretations, particularly when comparing estimates across different time points and regions. The EAPC method assumes log-linear trends over time, which may not capture non-linear disease pattern changes or sudden shifts due to external factors such as policy interventions, economic crises, or pandemic-related healthcare disruptions. Potential underreporting or misclassification of IHD cases, particularly in younger populations where symptoms may be atypical or attributed to other conditions, could affect trend estimates and absolute burden calculations. Despite these limitations, the study's comprehensive global scope, standardized methodology, and long-term temporal analysis provide robust evidence for policy development and resource allocation, while the identified patterns offer valuable insights for understanding the evolving epidemiology of early-onset ischemic heart disease globally.

These findings have important implications for clinical practice and public health policy. The identification of the 35–44 years age group as carrying the highest absolute burden suggests that targeted screening and intervention programs should focus intensively on this population. The substantial regional variations indicate that prevention strategies must be tailored to local epidemiological contexts and healthcare capabilities. The projected increasing burden underscores the need for enhanced primary care capacity, expanded cardiac rehabilitation services, and innovative approaches to reaching younger populations with cardiovascular risk factor modification programs (38, 39).

In conclusion, early-onset ischemic heart disease represents a growing global health challenge characterized by increasing incidence and prevalence but improving survival outcomes. The substantial sex and regional disparities identified highlight the need for targeted, equity-focused prevention strategies. While healthcare advances have improved outcomes for those developing disease, the projected substantial increases in absolute burden emphasize that population-level prevention must be prioritized to address this emerging epidemic effectively.

## Data Availability

Publicly available datasets were analyzed in this study. This data can be found here: https://vizhub.healthdata.org/gbd-results/.
