# Supplementary material for: Global, regional, and national burden of early-onset ischemic heart disease: trends and projections among adults aged 15–44 years from 1990 to 2046
Source: Front Cardiovasc Med. 2025 Aug 29;12:1653335. doi: 10.3389/fcvm.2025.1653335 (PMC12426116; doi:10.3389/fcvm.2025.1653335)
Supplement: Supplementary file 1 [file Table1.docx]

**
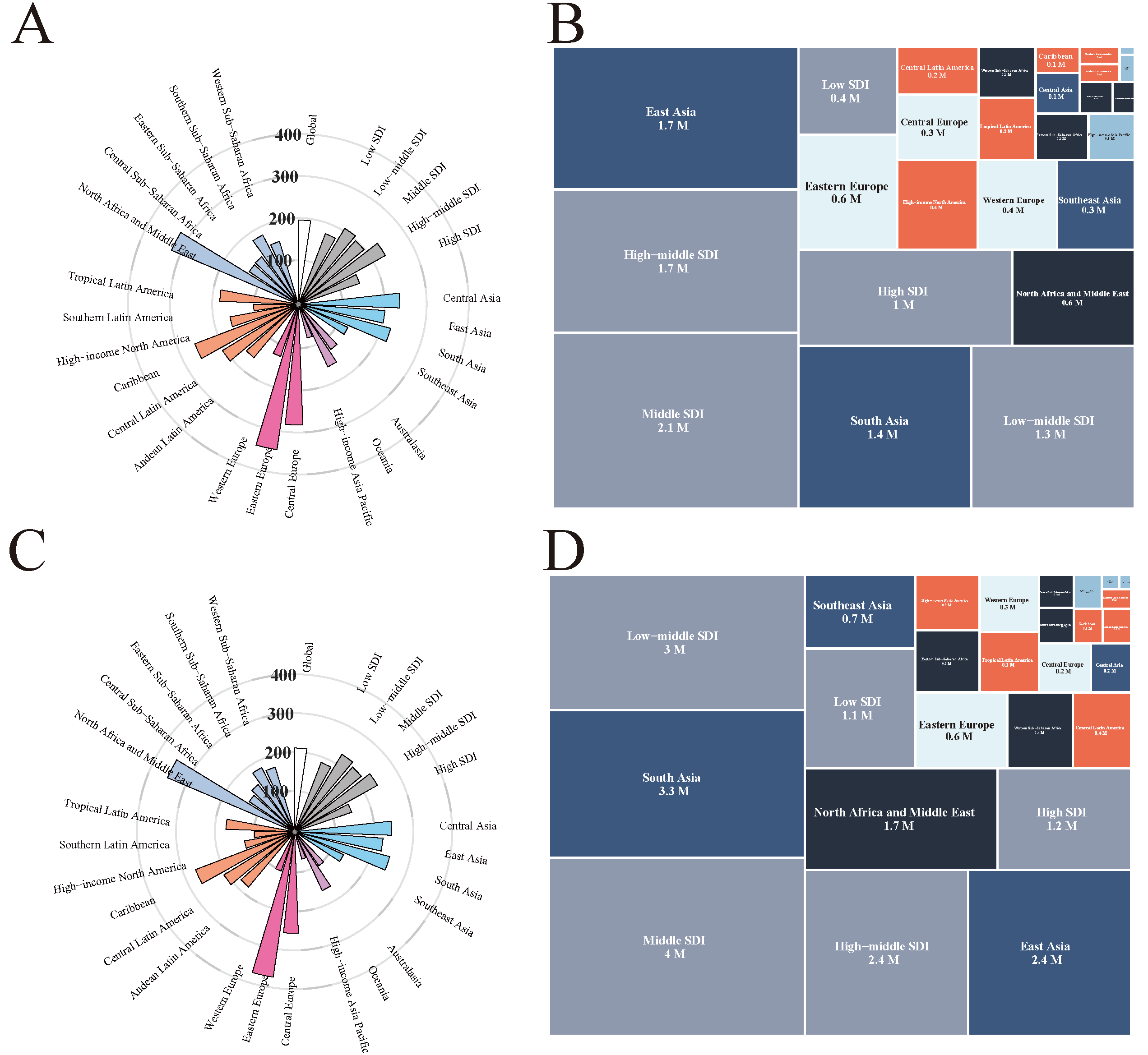
**

**Supplementary Figure 1.** Global epidemiological characteristics of early-onset IHD prevalence. (A) Regional distribution of ASPR of early-onset IHD in 1990: Radial bar chart showing the ASPRs across different regions and countries, expressed per 100,000 population. (B) Prevalent cases of early-onset IHD in 1990: Treemap visualization displaying the distribution of prevalent cases across different SDI regions, with values presented in millions. Middle SDI regions (2.1M), East Asia (1.7M) and High-middle SDI regions (1.7M) had the highest number of prevalent cases. (C) Regional distribution of ASPRs of early-onset IHD in 2021: Compared to 1990, showing the temporal changes in ASPRs across regions, expressed per 100,000 population. (D) Prevalent cases of early-onset IHD in 2021: Treemap showing the distribution of prevalent cases across different SDI regions in 2021, with Middle SDI (4M) and South Asia (3.3M) demonstrating substantial increases in case numbers.

IHD, ischemic heart disease; SDI, Socio-demographic Index; ASPR, Age-standardized Prevalence Rate; M, millions of cases.


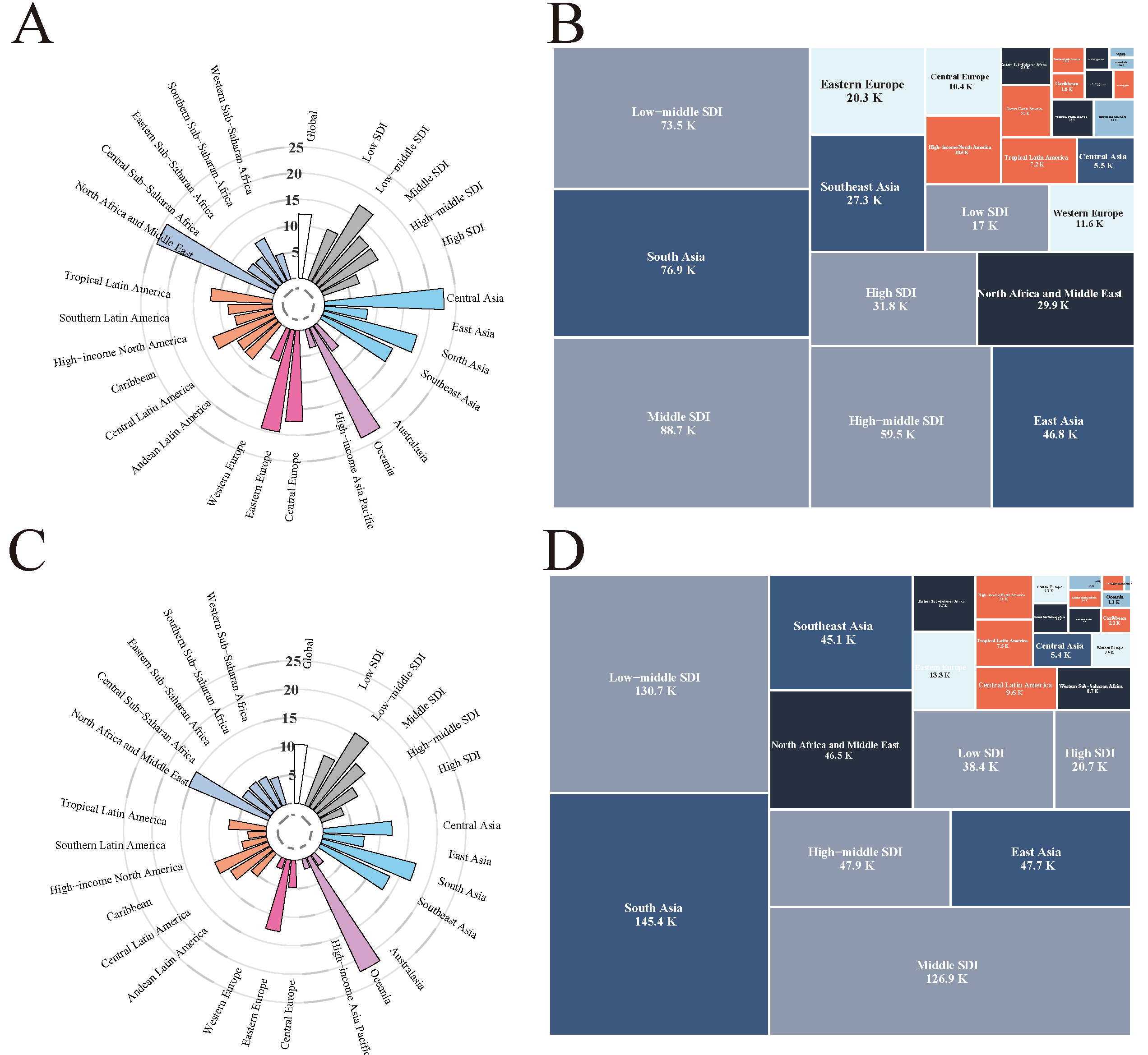


**Supplementary Figure 2.** Global epidemiological characteristics of early-onset IHD deaths. (A) Regional distribution of ASMRs of early-onset IHD in 1990: Radial bar chart showing the age-standardized mortality rates across different regions and countries, expressed per 100,000 population. (B) Deaths from early-onset IHD in 1990: Treemap visualization displaying the distribution of deaths across different SDI regions, with values presented in thousands. Middle SDI regions (88.7K) and South Asia (76.9K) had the highest number of deaths. (C) Regional distribution of ASMRs of early-onset IHD in 2021: Compared to 1990, showing the temporal changes in ASMRs across regions, expressed per 100,000 population. (D) Deaths from early-onset IHD in 2021: Treemap showing the distribution of deaths across different SDI regions in 2021, with South Asia (145.4K) and Low-middle SDI (130.7K) demonstrating substantial increases in death numbers.

IHD, ischemic heart disease; SDI, Socio-demographic Index; ASMR, Age-standardized Mortality Rate; K, thousands of deaths.

**
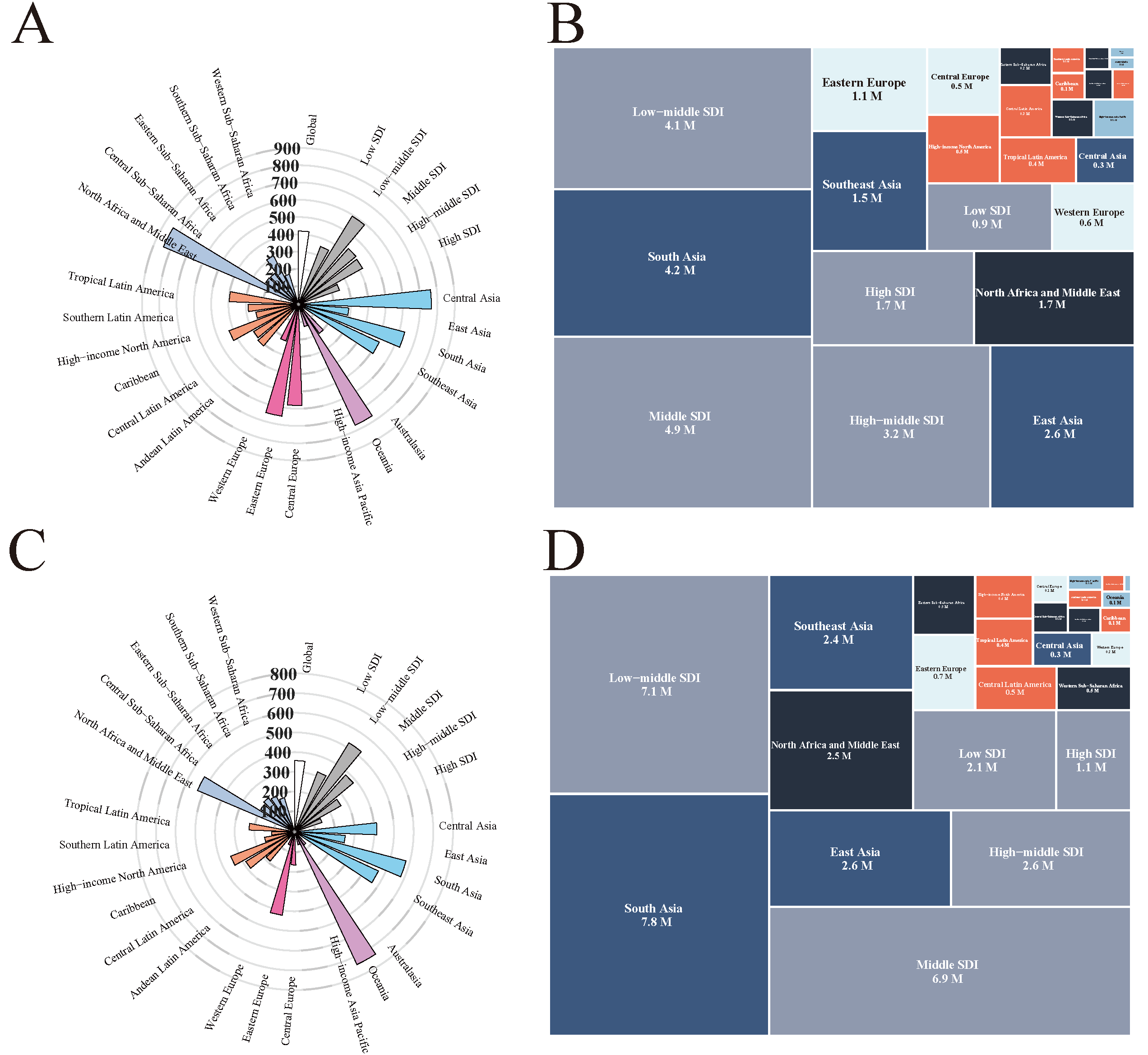
Supplementary Figure 3.** Global epidemiological characteristics of early-onset IHD DALYs. (A) Regional distribution of ASDRs of early-onset IHD in 1990: Radial bar chart showing the ASDRs across different regions and countries, expressed per 100,000 population. (B) DALYs from early-onset IHD in 1990: Treemap visualization displaying the distribution of DALYs across different SDI regions, with values presented in millions. Middle SDI regions (4.9M) and South Asia (4.2M) had the highest number of DALYs. (C) Regional distribution of ASDRs of early-onset IHD in 2021: Compared to 1990, showing the temporal changes in ASDRs across regions, expressed per 100,000 population. (D) DALYs from early-onset IHD in 2021: Treemap showing the distribution of DALYs across different SDI regions in 2021, with South Asia (7.8M) and Low-middle SDI (7.1M) demonstrating substantial increases in DALY numbers.

IHD, ischemic heart disease; SDI, Socio-demographic Index; DALYs, Disability-Adjusted Life Years; ASDR, age-standardized DALYs rate; M, millions of DALYs.

**
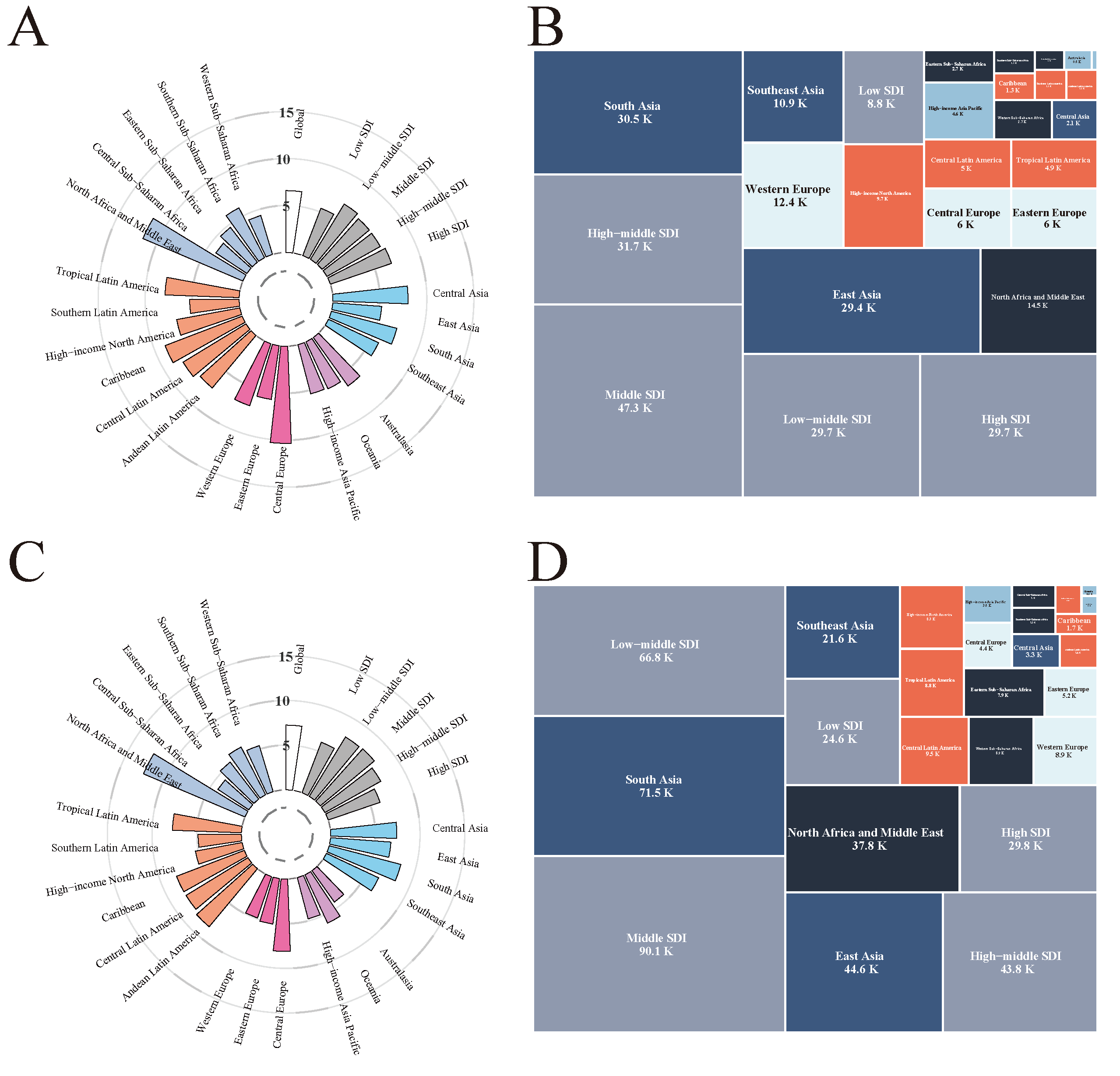
Supplementary Figure 4.** Global epidemiological characteristics of early-onset IHD YLDs. (A) Regional distribution of ASRs of early-onset IHD YLDs in 1990: Radial bar chart showing the ASRs across different regions and countries, expressed per 100,000 population. (B) YLDs from early-onset IHD in 1990: Treemap visualization displaying the distribution of YLDs across different SDI regions, with values presented in millions. Middle SDI regions (47.3K) and High-middle SDI (31.7K) had the highest number of YLDs. (C) Regional distribution of ASRs of early-onset IHD YLDs in 2021: Compared to 1990, showing the temporal changes in ASRs across regions, expressed per 100,000 population. (D) YLDs from early-onset IHD in 2021: Treemap showing the distribution of YLDs across different SDI regions in 2021, with South Asia (71.5K) and Middle SDI regions (90.1K) demonstrating substantial increases in YLDs numbers.

IHD, ischemic heart disease; SDI, Socio-demographic Index; YLDs, Years Lived with Disability; ASR, age-standardized rate; K, thousands of YLDs.


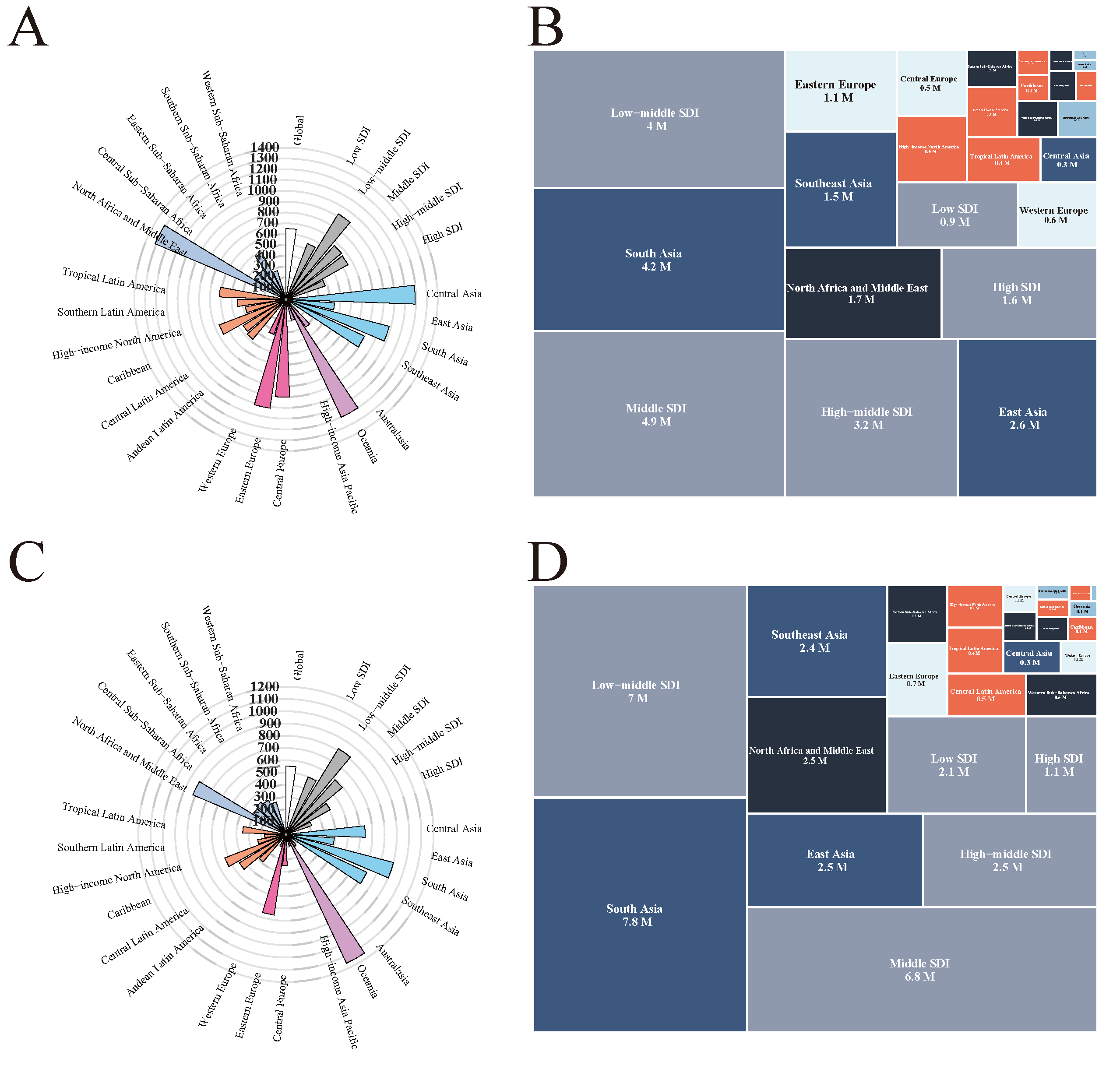


**Supplementary Figure 5.** Global epidemiological characteristics of early-onset IHD YLLs. (A) Regional distribution of ASRs of early-onset IHD YLLs in 1990: Radial bar chart showing the ASRs across different regions and countries, expressed per 100,000 population. (B) YLLs from early-onset IHD in 1990: Treemap visualization displaying the distribution of YLLs across different SDI regions, with values presented in millions. Middle SDI regions (4.9M) and South Asia (4.2M)had the highest number of YLLs. (C) Regional distribution of ASRs of early-onset IHD YLLs in 2021: Compared to 1990, showing the temporal changes in ASRs across regions, expressed per 100,000 population. (D) YLLs from early-onset IHD in 2021: Treemap showing the distribution of YLLs across different SDI regions in 2021, with South Asia (7.5M) and Low-middle SDI regions (7m) demonstrating substantial increases in YLLs numbers.

IHD, ischemic heart disease; SDI, Socio-demographic Index; YLLs, Years of Life Lost; ASR, age-standardized rate; M, millions of YLLs.


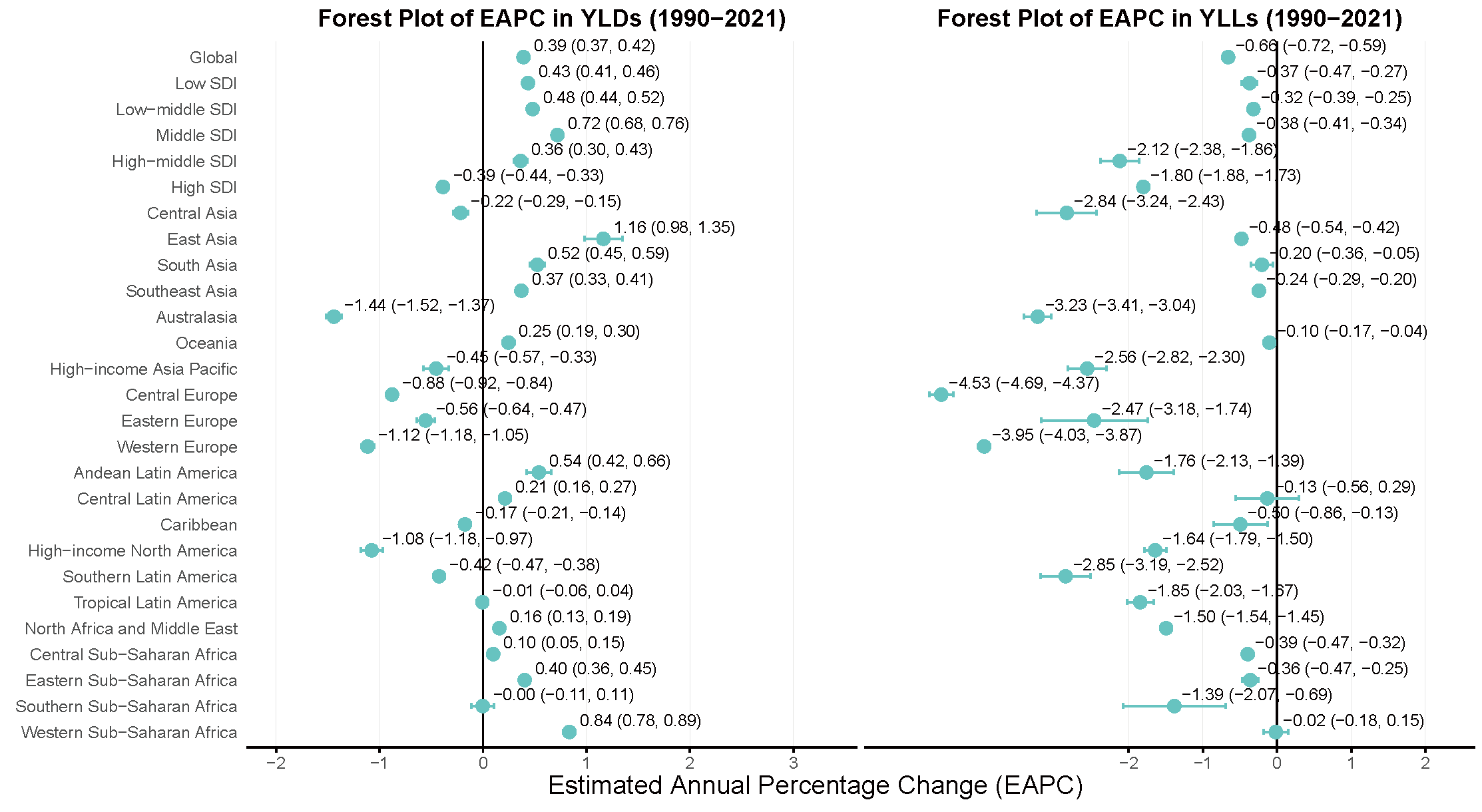


**Supplementary Figure 6.** Temporal trends in early-onset IHD burden by region and SDI level from 1990 to 2021. Forest plots showing the EAPC with 95% UIs for ASRs of YLDs and YLLs across different geographic regions and SDI levels. Positive EAPC values indicate increasing trends, while negative values indicate declining trends. The global EAPC was 0.39% (0.37-0.42%) for YLDs, and -0.66% (-0.72--0.59%) for YLLs.

IHD, ischemic heart disease; SDI, Socio-demographic Index; EAPC, Estimated Annual Percentage Change; UI, uncertainty intervals; ASR, Age-standardized Rate; YLDs, Years Lived with Disability; YLLs, Years of Life Lost.


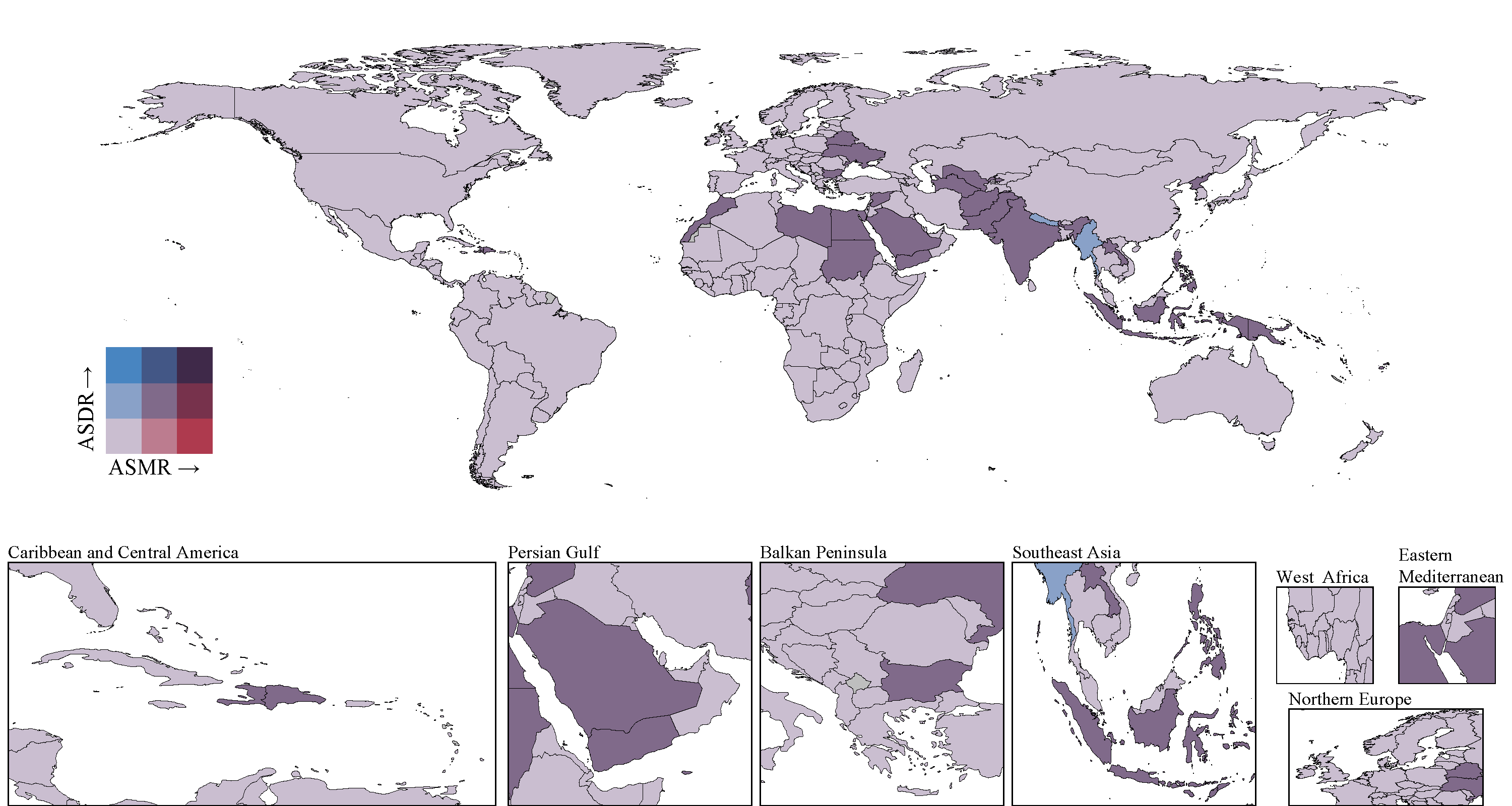


**Supplementary Figure 7.** Bivariate choropleth map showing the global distribution of ASMRs and ASDRs of early-onset IHD in 2021. Pacific Island nations demonstrated the most severe disease burden, with Nauru recording an ASMR of 78.09 per 100,000 and DALY rate of 2,610.35 per 100,000. Middle Eastern countries exhibited high mortality characteristics, with Egypt showing an ASMR of 29.27 per 100,000 and DALY rate of 1,040.66 per 100,000. Western European countries maintained low levels in both dimensions, with Sweden recording an ASMR of 0.82 per 100,000 and DALY rate of 30.32 per 100,000.

IHD, ischemic heart disease; ASMR, Age-standardized Mortality Rate; DALYs, Disability-Adjusted Life Years.

**
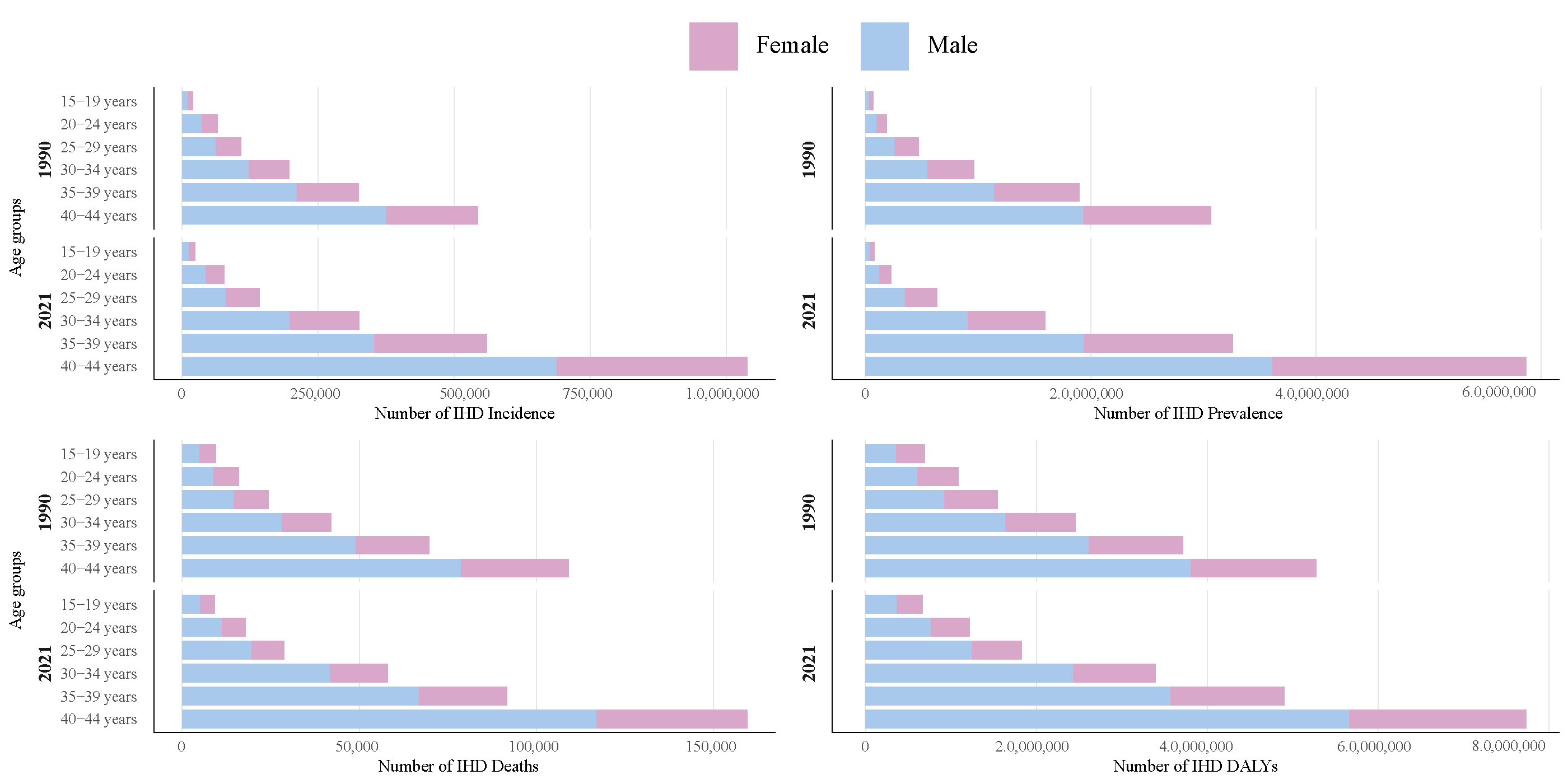
Supplementary Figure 8.** Global burden of early-onset IHD by age group and sex, 1990-2021. Each age group is represented by stacked bars showing female (pink) and male (blue) contributions for both 1990 and 2021. All measures demonstrate pronounced age gradients with the highest burden concentrated in the 40-44 years age group.

IHD, ischemic heart disease; DALYs, disability-adjusted life years.


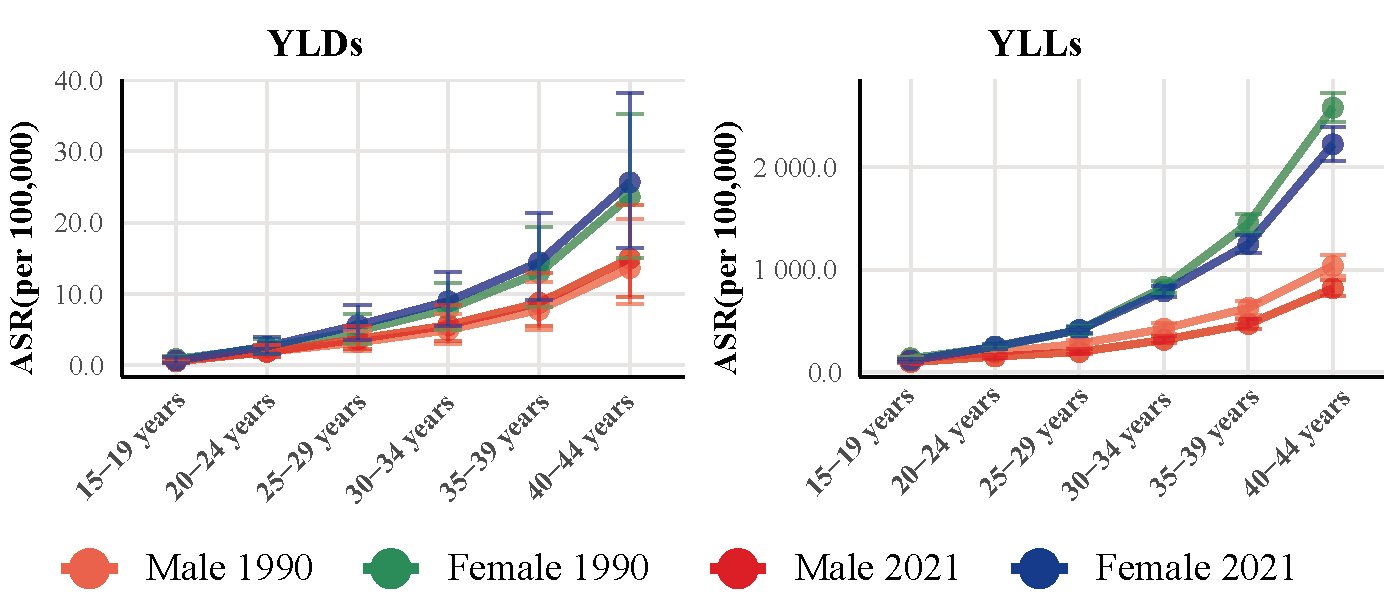


**Supplementary Figure 9.** Age and sex patterns of early-onset IHD burden in 1990 and 2021. ASRs with 95% UIs for YLDs, and YLLs by sex across six age groups from 15-44 years in 1990 and 2021. All measures demonstrated pronounced age gradients with exponential increases in older age groups.

IHD, ischemic heart disease; ASR, Age-standardized Rate; YLDs, Years Lived with Disability; YLLs, Years of Life Lost.


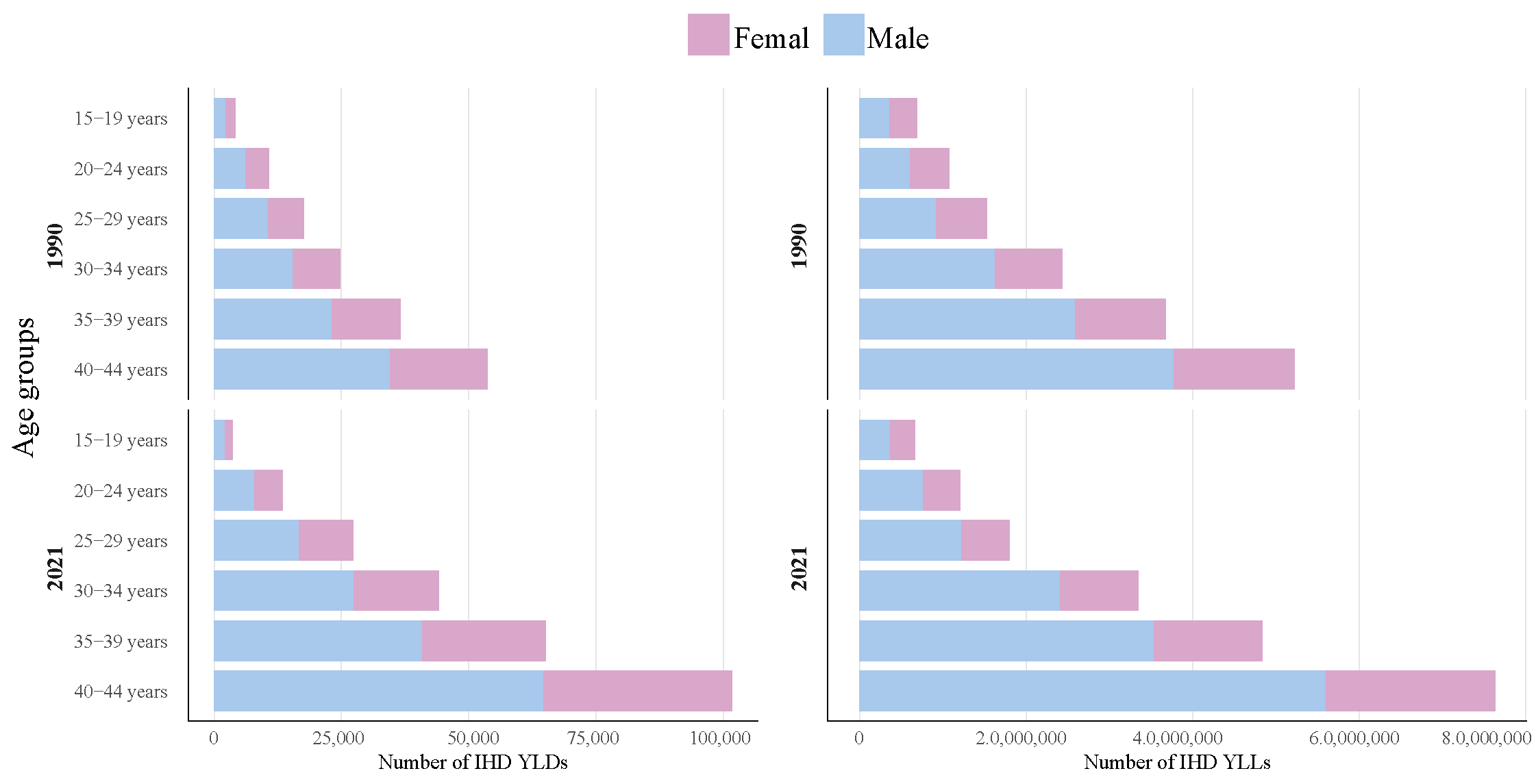


**Supplementary Figure 10.** Global burden of early-onset IHD by age group and sex, 1990-2021. Each age group is represented by stacked bars showing female (pink) and male (blue) contributions for both 1990 and 2021. All measures demonstrate pronounced age gradients with the highest burden concentrated in the 40-44 years age group.

IHD, ischemic heart disease; YLDs, Years Lived with Disability; YLLs, Years of Life Lost.


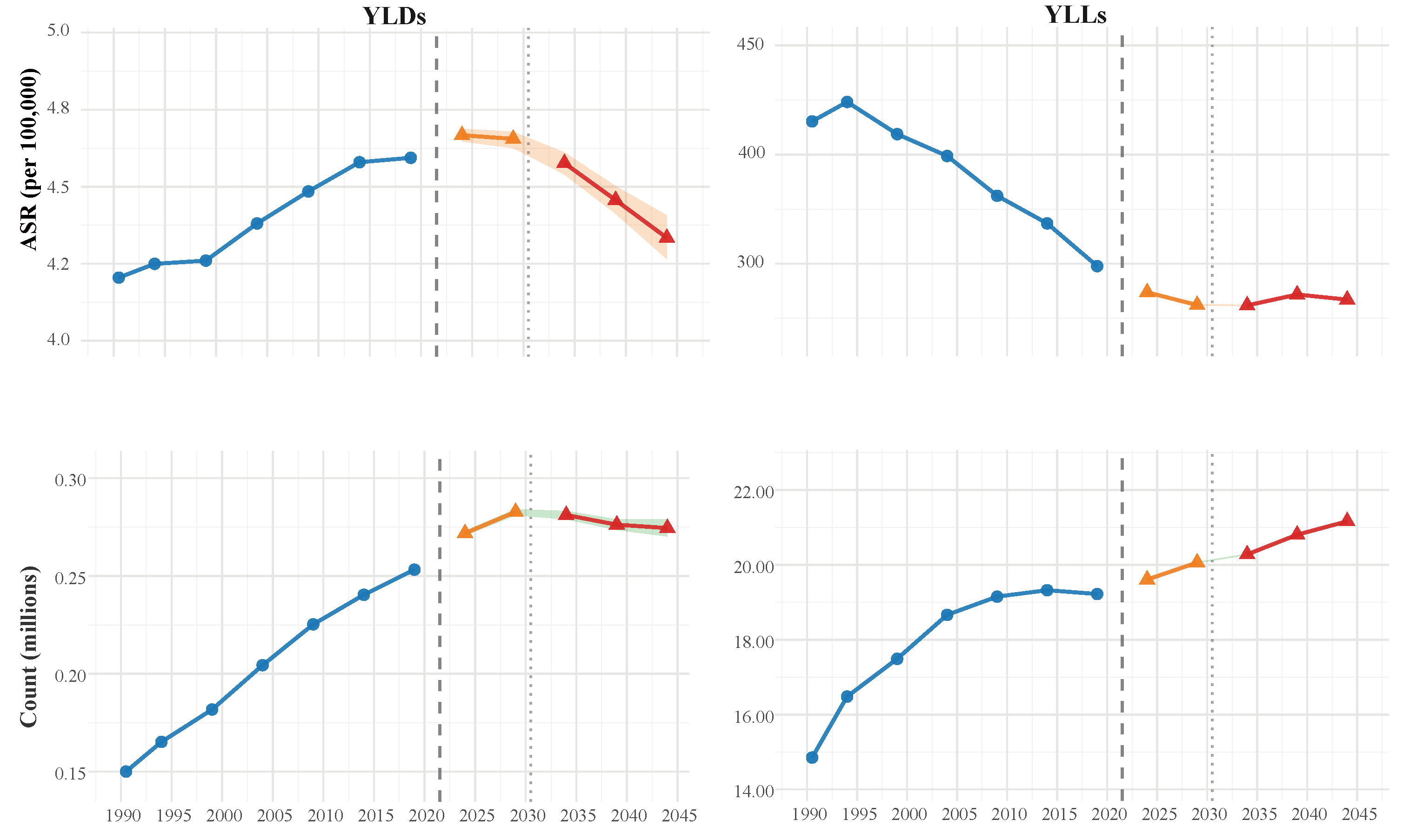


**Supplementary Figure 11.** Historical trends and future projections of early-onset IHD burden from 1990 to 2046. APC model projections showing historical observations (1990-2021) and future trends through 2046 for both age-standardized rates (upper panels) and absolute counts (lower panels). ASRs of YLDs are projected to continue rising to 4.3 (95% UI: 4.3-4.4) per 100,000 by 2046, with absolute incident cases reaching 0.27 (95% UI: 0.27-0.28) millions globally. ASRs of YLLs are projected to reach 333.5 (95% UI: 333-334) per 100,000 by 2046, corresponding to approximately 21.2 (95% UI: 21.1-21.2) millions worldwide. The vertical dashed line at 2021 separates historical observations from projections, with short-term (2022-2031) and long-term (2032-2046) projection periods indicated by different colors.

The model validation demonstrated satisfactory predictive performance with MAPE of 2.66% for ASRs of YLDs (correlation coefficient: 0.999), 4.32% for ASRs of YLLs (correlation coefficient: 0.997) using out-of-sample prediction (training: 1990-2011, validation: 2012-2021). The UIs shown in the projections capture both parameter uncertainty and natural variation in disease occurrence through bootstrap resampling methods.

IHD, ischemic heart disease; APC, Age-Period-Cohort; ASR, Age-standardized Rate; YLDs, Years Lived with Disability; YLLs, Years of Life Lost; UI, uncertainty intervals; MAPE, mean absolute percentage error; RMSE, root mean square error.
